# Moving beyond the lab: investigating empathy through the Empirical 5E approach

**DOI:** 10.3389/fpsyg.2023.1119469

**Published:** 2023-07-13

**Authors:** Alejandro Troncoso, Vicente Soto, Antoni Gomila, David Martínez-Pernía

**Affiliations:** ^1^Center for Social and Cognitive Neuroscience, School of Psychology, Adolfo Ibáñez University, Santiago, Chile; ^2^Department of Psychology, University of the Balearic Islands, Palma de Mallorca, Spain

**Keywords:** empathy, 5E approach, interbodily resonance, embodiment, MoBI, neurophenomenology

## Abstract

Empathy is a complex and multifaceted phenomenon that plays a crucial role in human social interactions. Recent developments in social neuroscience have provided valuable insights into the neural underpinnings and bodily mechanisms underlying empathy. This methodology often prioritizes precision, replicability, internal validity, and confound control. However, fully understanding the complexity of empathy seems unattainable by solely relying on artificial and controlled laboratory settings, while overlooking a comprehensive view of empathy through an ecological experimental approach. In this article, we propose articulating an integrative theoretical and methodological framework based on the 5E approach (the “E”s stand for *embodied, embedded, enacted, emotional, and extended perspectives of empathy*), highlighting the relevance of studying empathy as an active interaction between embodied agents, embedded in a shared real-world environment. In addition, we illustrate how a novel multimodal approach including mobile brain and body imaging (MoBi) combined with phenomenological methods, and the implementation of interactive paradigms in a natural context, are adequate procedures to study empathy from the 5E approach. In doing so, we present the Empirical 5E approach (E5E) as an integrative scientific framework to bridge brain/body and phenomenological attributes in an interbody interactive setting. Progressing toward an E5E approach can be crucial to understanding empathy in accordance with the complexity of how it is experienced in the real world.

## 1. Introduction

Empathy is a crucial component of the human emotional experience and is central to human social interactions. Empathy allows predicting and understanding the feelings, motivations, intentions, beliefs, and actions of others (Lamm et al., 2016; de Waal and Preston, 2017) and has been related to prosocial motivations and moral behavior, as well as to cruelty and immoral behavior (Decety and Cowell, 2018). Despite many definitions of empathy that exist, a general consensus is that empathy refers to our basic capacity to recognize, feel, and share another person’s world (Eklund and Meranius, 2021).

Given its importance to social cognition, in past decades there has been an increase in the empirical study in social neuroscience focusing on the neural bases of empathy. For instance, multiple fMRI studies have shown that the perception and/or imagination of another person in pain activates brain networks that are additionally involved in the firsthand experience of pain, supporting the idea of affective empathy (Lamm et al., 2011). In addition, similar cortical regions underlying the experience of emotions and sensations are also active when observing others’ emotions and sensations (Timmers et al., 2018). Other studies have reported specific brain responses in tasks that require cognitive evaluation, which has been associated with mentalization processes required to understand others (Samson et al., 2004; Saxe and Wexler, 2005). Furthermore, several studies have shown neural strategies for self-other distinction, which is a critical mechanism to distinguish between our experience and that of others (Decety, 2020). Based on this neuroscientific framework, the neural basis of empathy has been essential in understanding social cognition in clinical populations (Smith, 2009; Derntl et al., 2012), people with social vulnerability (Schulte et al., 2022), healthcare professionals (Decety, 2020), as well as the use of empathy training in the general population (Klimecki et al., 2014), and social interventions in educational and health contexts (Smith et al., 2017).

Regarding the methodological approach in the study of empathy, it has traditionally been examined in laboratory-based settings where participants are passively exposed to fixed stimuli (e.g., emotional faces) while their brain activity is recorded. This is the case of the study of either the neural response to the image of another’s pain (e.g., a pinprick to another’s finger) (Schulte et al., 2022) or the response to a story in a video (e.g., specific narratives requiring mentalization) (Klimecki et al., 2014). From this view, the main focus of empathy research is a segregated brain phenomenon that provides precise correlates for methodically decomposed fragments of elements (Ibanez, 2022). This methodology often prioritizes precision, replicability, internal validity, and control of confounds. However, fully understanding the complexity of empathy seems not achievable by solely relying on processing information in artificial and controlled laboratory settings, while neglecting an ecological approach.

Progress in social neuroscience has led to the development of experimental settings more concerned with empathy’s complex multidimensional nature. These insights have contributed to the contemporary understanding of empathy in social neuroscience, which conceives empathy as a multifaceted phenomenon influenced by various factors such as body state (movement, feeling, and posture) (Uithol and Gallese, 2015), context (Melloni et al., 2014), mode of interaction (Redcay and Schilbach, 2019), and subjectivity (first-person view) (Grice-Jackson et al., 2017). As a result of this progress, authors stemming from different disciplinary fields (e.g., neuroscience, psychology, and philosophy) have independently proposed understanding empathy focusing on brain–body responses, interaction dynamics, and phenomenological experience in more lived social interactions (Gallagher, 2012; Schilbach et al., 2013; Uithol and Gallese, 2015; Shamay-Tsoory and Mendelsohn, 2019; Levy and Bader, 2020; Fan et al., 2021).

Although these recent advances have allowed for a more complex understanding of empathy, this view still shows limitations in its methodological approach to understanding empathy in real-life situations. The primary limitation of contemporary social neuroscience is that, despite considering these innovative factors (body, context, interaction, and subjectivity), it often treats them as more or less independent processes. This approach neglects a holistic and integrated examination of their mutual influence and how they manifest in everyday situations, ultimately obscuring a more cohesive understanding. For example, the previously mentioned concept of the body could be expanded to a deeper understanding of embodiment, which involves the phenomenological experience of one’s own body as it interacts with the world and forms meaningful connections with others (e.g., de Jaegher et al., 2017). Consequently, studying empathy as it occurs in real life requires understanding through an integrative methodological framework, focusing on the physical interaction between embodied agents (physical bodies and experiences) within a specific context.

In this manuscript, we propose articulating such independent methodological approaches into an integrative methodological and theoretical framework based on the 5E approach. The 5E approach emphasizes the significance of the whole living body’s inter-corporeal interactions and phenomenological experiences for a deeper understanding of social cognition (Varela et al., 1991; Thompson, 2007; Colombetti, 2014; Newen et al., 2018; Pérez and Gomila, 2022). Bearing this in mind, we will explore how an integrated study of empathy can be incorporated into scientific research, enabling the integration of embodied, embedded, inter-corporeal, and phenomenological aspects.

This article is divided into four sections. The first section of this article will analyze the four primary insights derived from recent developments in empathy neuroscience and discuss the limitations that must be addressed to advance toward a more ecological study of empathy. The second section begins by laying out the main theoretical contributions of the 5E approach in the empathy study. In addition, we argue a shift toward understanding empathy as an embodied, embedded, inter-corporeal, and complex experiential phenomenon. Then, and in coherence with these assumptions, we will show in the third section a scientific methodology highlighting the use of wireless technologies in natural contexts, the interpersonal dynamics in interbodily paradigms, and the description of a rigorous method to study lived experience. Finally, in the fourth section of the manuscript, we will show a scientific method to foster integration between the embodied, embedded, inter-corporeal, and phenomenological dimensions of empathy based on the 5E approach. For this, an Empirical 5E approach (E5E approach) is proposed as the method that allows the study of neurophysiological attributes (third-person view) and the phenomenological experience (first-person view) in an inter-corporeal and interactive setting of two or more embodied agents (second-person view).

## 2. Paving the way for a comprehensive understanding of empathy: advances and limitations in social neuroscience

Recent advances in social neuroscience have highlighted empathy as a multidimensional phenomenon that is influenced by sensory-motor processes, contextual and environmental factors, and individual subjective experiences (e.g., Melloni et al., 2014; Lamm et al., 2016; Riečanský and Lamm, 2019). These advances have been primarily studied in controlled laboratory settings (e.g., observation of artificial pain stimuli), allowing the experimental manipulation (e.g., first-person experience of pain) to isolate the mechanisms of empathy (e.g., shared brain activation between one’s own pain and that of others) (Rütgen et al., 2015a,b). Despite the wealth of knowledge accumulated about isolated phenomena through laboratory studies, this success has become a double-edged sword. While the confined lab-setting has provided valuable insights into specific aspects of empathy, it has also posed challenges in comprehending how empathy functions in everyday situations, resulting in a “golden cage” limiting our ability to understand cognition outside the laboratory setting (Ibanez, 2022).

**Figure 1:**
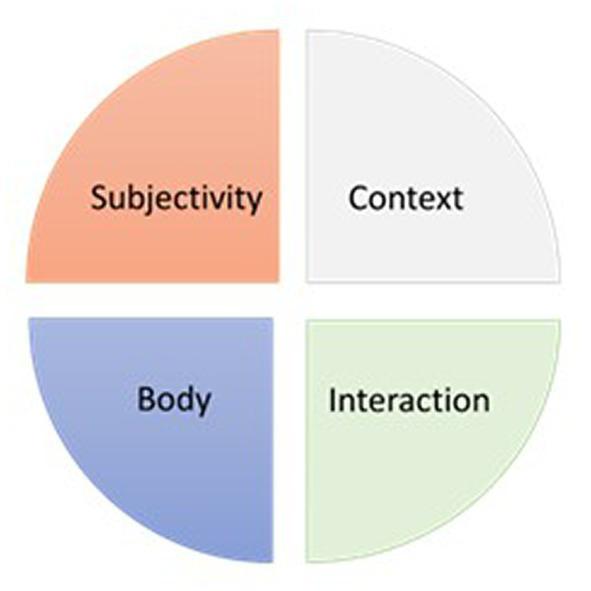
Empathy research in the field of social neuroscience has shed light on the multifaceted nature of empathy, emphasizing the mutual influences between empathy and various factors such as the body, interaction, subjectivity, and context. However, these influences have often been examined in isolation, prioritizing experimental control over ecological validity and comprehensive understanding.

In everyday situations, the body—both its physical and experiential aspects—is fully engaged through posture, movement, voice, and feelings within a multisensory and dynamic natural context (Shamay-Tsoory and Mendelsohn, 2019; Fan et al., 2021). Additionally, the body interacts with other beings who are also shaped by their historical relationship. While these influences have been recognized in isolation in laboratory research (Figure 1), there is still a need to understand empathy in real-life situations.

In this section, we explore how contemporary advances in neuroscience have discovered the influences on the empathy of the body (1.1), context (1.2), interaction (1.3), and subjectivity (1.4). Additionally, we address the inherent limitations of its methodology in understanding empathy in the real world. Understanding both the contributions and limitations in contemporary social neuroscience could pave the way for designing research that considers the mutual influences of these elements.

### 2.1. Influence of body in empathy

Research on the role of the body in empathy has been a prominent focus in the field over the past few decades. The importance of the body in empathy is widely accepted among social cognition researchers (Uithol and Gallese, 2015; de Waal and Preston, 2017; Fuchs, 2017; Riečanský and Lamm, 2019). Studies conducted in a well-controlled laboratory setting have examined multiple levels of the nervous and musculoskeletal systems’ responses to the pain of others (Riečanský and Lamm, 2019), and have utilized body manipulations such as muscle blocking (Oberman et al., 2007; Borgomaneri et al., 2020), and posture changes (Weineck et al., 2020; Zloteanu et al., 2021), revealing intriguing findings. For instance, studies have shown that the sensory-motor regions of the nervous system that are active in response to first-hand pain also become active in response to the pain of others (Riečanský and Lamm, 2019). This bodily resonance occurs at multiple levels of the nervous system, and numerous studies have shown postural (Gea et al., 2014; Lelard et al., 2019), muscular (Lamm et al., 2008; Hess and Bourgeois, 2010; Light et al., 2015), physiological (Eisenberg et al., 1988, 1991), and kinematic correlates in empathy.

Furthermore, research suggests that the body’s involvement in empathy is not limited to being affected by other beings, as it also plays an active role in the empathic response through action (e.g., Borgomaneri et al., 2020). The body’s expression also allows the tendency to act toward others, and this bodily expression can affect both the agent’s own emotions and the emotional recognition of others (Niedenthal, 2007). For instance, when emotional facial expressions do not match emotional body postures, emotional recognition is impaired (Meeren et al., 2005). Additionally, when facial muscles are blocked using an unrelated task (biting on a pen) or Botox injections the emotional recognition of another’s expression is hampered (Oberman et al., 2007; Neal and Chartrand, 2011; Borgomaneri et al., 2020). This suggests that empathy is a dynamic process that involves both the perception and action of emotions through the body.

Complementing the role of bodily resonance in empathy, research on body posture reveals its influence on how individuals perceive and react to others, as well as their own emotional behavior. For instance, adopting an open posture increases accuracy in detecting lies and affects gazing behavior (Zloteanu et al., 2021).

Another important body factor influencing empathy is the ability to detect internal bodily changes, known as interoception (Fukushima et al., 2011; Grynberg and Pollatos, 2015; Shah et al., 2017). For instance, Grynberg and Pollatos (2015) reported that interoception is related to both neural responses of empathy and self-report measures in traditional settings. In summary, insights from social neuroscience have underscored the fundamental role of brain–body coupling in understanding empathy.

While insights from previous research have allowed for a shift from viewing empathy as a purely brain-based phenomenon to recognizing its dependence on corporeality and physiology, the methodologies traditionally employed in studies do not always allow for the full involvement of the body in the process. Additionally, the body is often studied outside of its ecological context, which may limit the extent to which researchers can capture the complexities of embodied empathy (Shamay-Tsoory and Mendelsohn, 2019; Stangl et al., 2023). Given the essential role of the body in empathy, the minimal participation of the body in confined lab settings poses a methodological limitation that could affect our understanding of empathy in more naturalistic contexts.

Given the restrictions in body movement required by modern brain imaging techniques such, such as functional magnetic resonance imaging (fMRI), positron emission tomography (PET), magnetoencephalography (MEG), and static electroencephalography (EEG), studies investigating brain responses to others’ suffering have often minimized the body’s influence on the empathic brain process. In these paradigms, participants are typically required to remain motionless during signal acquisition, which limits the extent to which the body can be fully engaged in the empathic experience (e.g., Lamm et al., 2011). For instance, the participants are asked not to perform facial or body expressions, avoid speaking, and remain unnaturally still when observing another’s pain (e.g., Lamm et al., 2011). Moreover, most brain imaging techniques require large and bulky sensors making the majority of empathy experimental paradigms stationary (Gramann et al., 2011).

When it compared this lab approach with the natural interactive context, the influence of posture, bodily expression, bodily feeling, and voice, shows differences between those approaches. Likewise, in real life, the agent has free bodily expression and a tendency to act toward others (e.g., by freeze or approximation responses) (Gea et al., 2014; Lelard et al., 2017, 2019). Additionally, in real-life interactive contexts, the dynamics of the body are influenced by others, resulting in a complex cycle of bodily coordination.

Empirical studies conducted in laboratory settings have demonstrated how restrictions on body movement, the use of unnatural postures (such as lying down, which is required in fMRI studies), and limitations on interbodily synchrony can affect the underlying processes of empathy. Firstly, empirical studies have shown that constraints to body movement and position decline emotion recognition accuracy (Reed et al., 2020) In addition, restricted movement and unnatural posture can negatively impact the quality of bodily sensations and interoception in a social context. Weineck et al. (2020) further showed that the use of unnatural body posture strongly affects interoception.

Secondly, direct evidence comparing unnatural posture in regard to the experimental tasks performed (e.g., performing emotional detection tasks while assuming a supine position in an fMRI scanner) has been criticized by its influences on perception, behavior, physiology, performance, and brain activity (for review see Thibault et al., 2014, 2016). For example, greater blood flows in both visual and cerebellar cortices have been shown when standing erect compared to lying supine (Ouchi et al., 1999, 2001). Likewise, upright versus supine posture has been shown to increase generalized high-frequency oscillatory activity in resting state EEG and MEG (Thibault et al., 2014, 2016). Collectively, these studies outline the critical role of the use of natural postures in the study of social cognition, and more specifically, in empathy.

Thirdly, non-coordinating movements with others have been shown to have a negative impact on empathy, affiliative attitudes, and behaviors. Meta-analytic evidence suggests that experimentally manipulated synchronous action has a significant influence on social cognition (Mogan et al., 2017). This dynamic interpersonal coordination is absent in a traditional lab setting due to the absence of another being.

The evidence presented in this section suggests that the brain/body dynamics of empathy are coupled with bodily behavior and bodily behavior shapes those dynamics. Also, empathy is a dynamic process of two or more bodies coordinated in a shared context. However, most studies on empathy and the role of the body in empathy lack rich social interaction in an ecological context and tend to overlook the natural involvement of the body. This limitation presents an opportunity to improve the ecological understanding of empathy by including the full involvement of the body in empathic interactions, and by deepening our understanding of the role of the body in broader concepts.

### 2.2. Influence of context in empathy

Over the years, there has been a significant emphasis in the field on studying the impact of contextual factors on empathy (Uithol and Gallese, 2015; de Waal and Preston, 2017; Fuchs, 2017; Riečanský and Lamm, 2019). Studies conducted in well-controlled laboratory settings have examined the influence of various contextual factors through context manipulation and comparisons between participants from different socio-cultural backgrounds (e.g., group membership). These studies have enabled researchers to uncover the intricate relationship between context and empathy (for review see Melloni et al., 2014). Consequently, numerous authors concur on the embedded nature of empathy (for review see, Hein and Singer, 2008; Bernhardt and Singer, 2012; Kennedy and Adolphs, 2012). This nature demonstrates empathy adaptability, enabling individuals to adjust their responses according to the specific requirements of the prevailing situation (Bernhardt and Singer, 2012).

Several studies from traditional paradigms (e.g., picture or video-based tasks) reveal how context modulates the brain responses to empathy (Akitsuki and Decety, 2009; Cheng et al., 2017). For example, distinct behavioral ratings and brain activation patterns have also been reported among medical professionals when perceiving others’ pain in a hospital setting compared to at home, using a picture-based paradigm (Cheng et al., 2017). Furthermore, Iacoboni et al. (2005) found a more robust activation of “mirror neurons” when participants observed actions carried out with social intent (e.g., offering a cup of tea) as opposed to those with individual intent (e.g., picking up a cup for oneself).

In addition, by manipulating the group membership, individuals showed higher subjective empathy-related ratings, and electrodermal activity (EDA) toward stimuli that were phylogenetically more similar to humans (e.g., primates vs. birds) (Rae Westbury and Neumann, 2008). Likewise, A study by Xu et al. (2009) found that racial group membership can also impact empathic processes. Overall, these results demonstrate that empathy responses can be significantly influenced by the participant context, as well as the broader socio-cultural context in which individuals are embedded.

Utilizing static images and video clips has afforded researchers significant control and flexibility, enabling them to uncover key insights into empathy, including its connections to prosocial actions, moral behavior, and action understanding. However, to further our knowledge of empathy in real-life situations, this approach needs to improve its ecological validity. The disparities between the stimuli and context highlight a fundamental distinction from real-life situations.

Commonly, in traditional settings, the nature of stimuli and the context are mostly artificial stimuli (e.g., pictures and videos) focusing on 1–2 sensory modalities (e.g., visual modality) (e.g., Lamm et al., 2011). In addition, the presentation of another being is mostly from a disembodied being that is presented as a part of the body (e.g., faces or fingers) (e.g., Lamm et al., 2011). Nevertheless, in real-life interaction, we are affected by multisensorial modalities such as the other’s tone of voice, gestures, body postures, temperature, and context in a dynamic fashion (Shamay-Tsoory and Mendelsohn, 2019). Recent studies suggest that utilizing more natural stimuli can have a significant impact on participant engagement and their corresponding neurophysiological responses. For instance, viewing dynamic faces displaying various expressions can lead to better recognition of emotions, facial muscle activity in observers, and higher ratings of the faces as emotional compared to viewing static facial images (Richoz et al., 2018). Furthermore, empirical evidence demonstrates that body expression has a significant contextual impact on facial emotion perception as assessed by EEG (Rajhans et al., 2016; Treal et al., 2021). Moreover, when observing a virtual character expressing pain, participants displayed heightened empathic responses to the virtual individual’s suffering when natural postural oscillation was present, compared to a static condition (Treal et al., 2021). Likewise, moving beyond the picture or movie-based paradigm, virtual reality (VR) enables researchers to study empathy by simulating complex social situations and maintaining control of the environmental stimuli (Parsons et al., 2020). For instance, previous studies have used VR to study empathy and have shown more effectiveness than video in eliciting empathy toward refugees (Schutte and Stilinović, 2017) and influencing pro-environmental behavior (Nelson et al., 2020).

Overall, by utilizing more naturalistic stimuli, researchers can potentially increase participant engagement and gain a deeper understanding of the underlying neurophysiological mechanisms involved in empathy. Therefore, given the contextual nature of empathy, its research would benefit to transit to a more ecological approach that takes into account the real-life situations and complexities that shape empathic responses and experiences.

### 2.3. Influence of physical interaction with another in empathy

Most of our social experiences involve engaging in mutual interactions. However, despite the importance of social interaction in our daily lives, research on the empathy processes underlying these interactions has often been conducted in non-interactive settings. In such settings, a passive form of interaction is employed, wherein individuals observe a computerized being without actively engaging with it. They rely on physically limited information, such as a hand or face, to infer the mental states of others without direct interaction (e.g., Lamm et al., 2011).

There is accumulated evidence that social cognition and underlying neural-body mechanisms are fundamentally different between paradigms with a mere observation and paradigm during active interaction in computerized tasks (Schilbach et al., 2013). Accordingly, a methodological approach called the “second-person approach” was proposed to study social interaction (Schilbach et al., 2013). Those experimental settings require at least individuals that feel engaged with another and/or individuals who participate in social interaction (Redcay and Schilbach, 2019). Recent studies using the 2p (second-person) approach have shed light on the neural mechanisms underlying important aspects of social interaction, such as mutual engagement and joint attention (Redcay and Schilbach, 2019). However, many of these studies have been conducted in traditional laboratory settings using controlled and predictable tasks, such as two participants lying in separate MEG or fMRI (Hirata et al., 2014; Sperduti et al., 2014), and studies of human-avatar or human-computer interactions (Schilbach et al., 2006; Cohen et al., 2009). Also, some studies have used VR, which simulates realistic scenarios that may be challenging to create in real-life (Parsons et al., 2020). Such settings do not capture the dynamic nature of natural social interactions (Redcay and Schilbach, 2019; Martínez-Pernía et al., 2023a).

Recent studies have highlighted the importance of naturalistic settings in exploring empathic interactions (Goldstein et al., 2017; Schwartz et al., 2022). For example, Schwartz et al. (2022) found that live face-to-face interaction between mothers and children showed significantly more brain-to-brain synchrony compared to technologically-assisted communication. Similarly, another study reveals that the mere co-presence of another being generates automatic physiological coordination between agents (Golland et al., 2015). Overall, those studies suggest that human co-presence involves unique neurobiological processes and highlights the need to explore empathic interaction in naturalistic settings. The underlying mechanisms active during this coordinated interaction are often absent (e.g., picture-based task) or diminished (interaction in a computerized task) in the classical laboratory.

**Figure 2:**
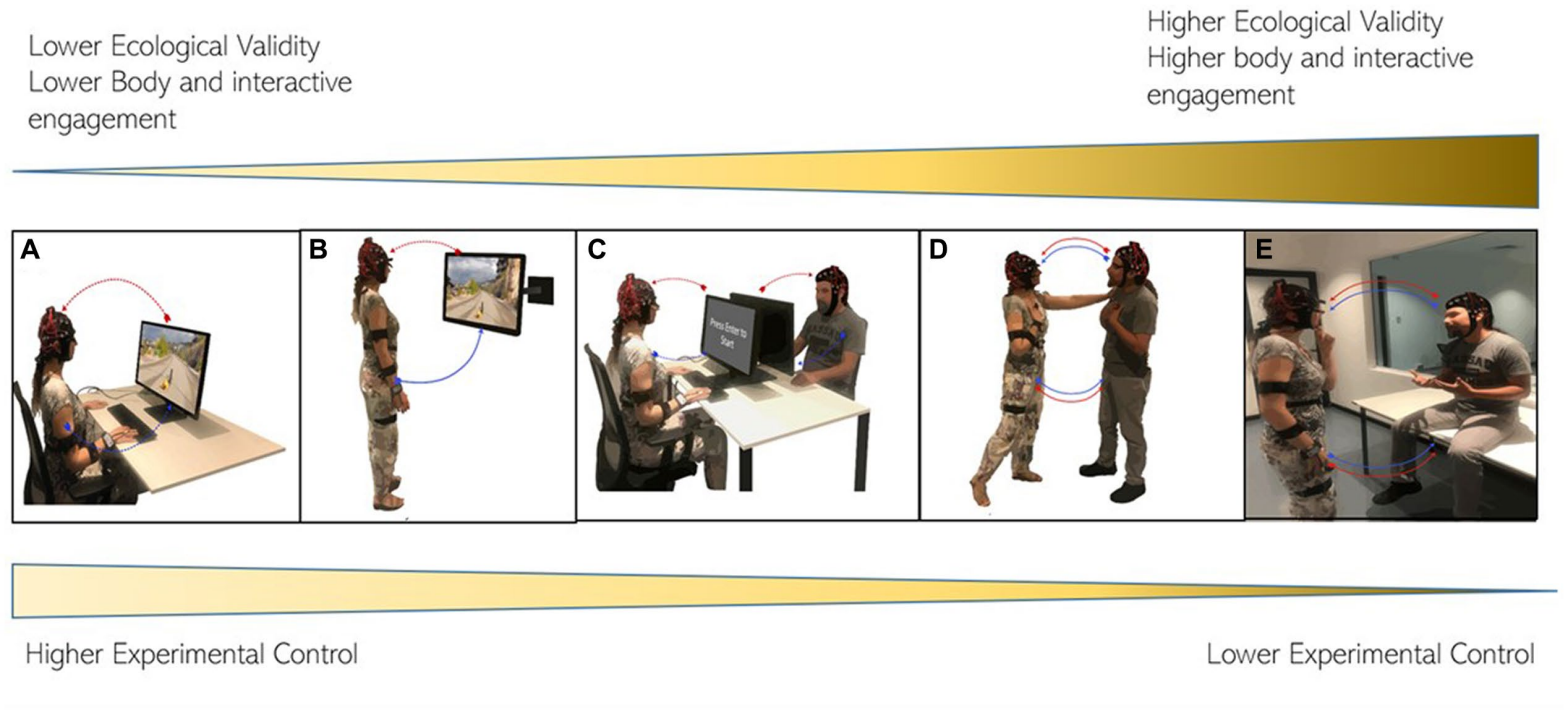
Depicts different experimental settings used in social neuroscience. **(A)** Shows a traditional computerized task (e.g., picture or movie-based). **(B)** Shows an upgrade to the computerized task incorporating the bodily state (upright posture). **(C)** Shows the active interaction in a computerized task. **(D)** Illustrates a natural active interaction. **(E)** Represents an interaction in a real-world context. This image E shows the possibility of being fully affected by another being in a shared context and the possibility to act in a free form. In methodological terms, in this figure shows different levels of ecological validity and experimental control. More naturalistic settings prioritize ecological validity over experimental control.

Taken together, these findings illustrated the importance of an interactive lab setting in the research of empathy (Figure 2 summarizes the different methodological approaches in the empathy study according to their naturalistic setting). Therefore, the understanding of empathy would benefit from the translation to a more real interactive setting.

### 2.4. Subjectivity in empathy

The subjective experience is crucial in understanding empathy as it provides insights into the emotional and cognitive processes involved in sharing others’ feelings (Decety and Cowell, 2014). Examining subjectivity helps researchers comprehend how individuals perceive, interpret, and respond to others’ emotions, leading to comprehensive empathy models and interventions to enhance empathic abilities (Decety and Jackson, 2004; Zaki and Ochsner, 2012). There are two basic approaches currently being adopted in empathy research to explore quantitatively the subjective experience. One is a self-report assessment in the experimental research that required reporting the experience (in a verbal or behavioral response) when a specific empathic stimulus is provided, e.g., a finger being pricked with a needle (e.g., Lamm et al., 2011) or an agent narrating a story (e.g., Klimecki et al., 2014). These self-reports have been useful to validate empathy paradigms and relate the neurophysiological and behavioral findings with the subjective experience (e.g., Klimecki et al., 2014; Timmers et al., 2018). Another is self-report based on scale questionnaires to describe their past experiences (e.g., Interpersonal Reactivity Index -IRI-). This has been useful to compare neurophysiological responses between different clusters based on the traits of the participants (e.g., high empathy trait vs. low empathy trait) (e.g., Goldstein et al., 2017).

Both approaches are defined as weak introspective methods (Olivares et al., 2015). They explore subjective experience based on its intellectual interpretation and focus on the explicit or reflective aspect of the experience, that is, the mental content (Olivares et al., 2015; Hamilton et al., 2019). For instance, when participants are asked to evaluate the quantity of empathy-compassion, valence, intensity, and/or arousal on a Likert-based scale, they use an introspective gesture to reflect and judge their experience through an intellectual and rational perspective (Olivares et al., 2015).

Despite the important use of the introspective self-report method in empathy research, several limitations have been described. Those limitations may be classified in terms of the lack of complexity and methodological biases of the subjective information collected. Concerning the complexity bias, the introspective method oversimplifies the subjective experience of empathy, reducing it to a mere degree or quantity (Petitmengin, 2006; Olivares et al., 2015). This approach may overlook the nuances and complexities of how the empathic experience unfolds, as well as how it is experienced in the context of interpersonal interactions (Petitmengin, 2006; Olivares et al., 2015). This is qualitatively different from a deep examination of subjectivity, which moves out from interpretation, judgment, and reflection about the experience. A deep examination of the complexity of subjective experiences considers their multidimensional dynamics (bodily, affective, visuospatial perspective, attentional focus, and so on) of the implicit or pre-reflective part of the experience (Petitmengin, 2006; Olivares et al., 2015). Empirical findings presented by Depraz et al. (2017) have pointed to phenomenological experience as multidimensional, complex and fluctuating in nature. This multidimensionality is described as a subtle characterization of bodily, affective, attentional, and internal dialogue aspects while the fluctuating nature refers to a change of this multidimensional state in a succession of instants. The absence of those experience’s complex dimensions in the self-report questionnaires does not allow a clear exploration of the experience being evaluated in experimental settings. On the other hand, it is important to address the potential methodological bias in contemporary studies that gather first-person views, as the very process of collecting such data can induce a subjective state. This introduces a potential bias when researchers assume that participants are fully aware of the predetermined questions about their emotional state (e.g., distress, warmth) or empathic perception (e.g., valence, arousal) to which they are being exposed (Hurlburt and Heavey, 2015). Furthermore, these pre-set questions of an experiential domain reveal investigators’ previous theoretical assumptions about the nature of empathic experience and thus prevent knowing the participant’s subjective in their words (Colombetti, 2013). Another methodological bias of introspective methods is the underestimation or overestimation of how they feel about the affective reactions of others (Mascaro et al., 2020). Further, the retrospective measures tend to describe judgments and beliefs about oneself, which in empathy can be problematic, considering that an empathic person is related to positive social aspects, such as the ability to generate closer bonds (Morelli et al., 2017).

Overall, this sub-section illustrates that the complexity of subjectivity cannot be fully explored by relying on classical self-reporting questionnaires alone. Given the rich insight that comes from the in-depth examination of lived experience, empathy research would benefit from integrating the phenomenological experience into the paradigm.

## 3. Empathy from 5E approach: an integrative proposal based on a theoretical progression

In the previous section, we highlight the contributions of social neuroscience and its main limitations that need to be addressed to transit to an interactive and naturalistic study of empathy. Based on these limitations, the current challenge facing the study of empathy in the real-life and interactive context is the full incorporation of the construct of embodiment in scientific research, that is, empathy is an embodied, intercorporeal, and experiential phenomenon embedded in a natural and social context. Authors of the 5E approach have theoretically addressed this incorporation, offering a new perspective on empathy as an interbodily interactive process within natural contexts. In this section, we explore the theoretical refinements put forth by this approach. Afterward, we discuss an empirical methodology that takes a mobile multidimensional approach within an interbodily natural context, adhering to the aforementioned premises. This represents a significant advancement toward overcoming the limitations of current empathic research.

Drawing on studies from classical neuroscience and other disciplines (philosophy, psychology, sociology, anthropology), a novel perspective of cognition has been proposed, namely 5E. This approach proposes that cognition is embodied, embedded, enacted, emotional, and extended (Varela et al., 1991; Thompson, 2007; Colombetti, 2014; Newen et al., 2018; Pérez and Gomila, 2022). Embodied empathy emphasizes that comprehending others is rooted in possessing a body with diverse sensorimotor capabilities, which are situated in a broader biological, psychological, and cultural context (Varela et al., 1991; Tanaka, 2015). Here, the concept of embodiment is further elaborated through the 5E approach, in which the body is no longer understood in isolation through the brain or neuro-physiological processes. Instead, the body is seen as a dynamic entity where the neurophysiological processes (living body) and also processes of experiencing (lived body) are intertwined and interact dynamically with the environment (Gallagher, 2015; Fuchs, 2020; Lindblom, 2020). The embedded aspect of 5E implies that a person’s body is always situated in an environment, and that empathy is shaped by the individual’s relationships and interactions with their physical and sociocultural surroundings. (Melloni et al., 2014; Newen et al., 2018). Enacted empathy refers to the active process that arises from the dynamic interaction between embodied agents and their environment, where meaning is brought forth through intimate and active engagement (Varela et al., 1991; Fuchs, 2017). In other words, enactive empathy is a process that emerges from the ongoing, dynamic and adaptive relationship between the mind, body and context (Varela et al., 1991). In terms of the emotional aspect, empathy is fundamentally an affective experience involving resonance and understanding on an affective level (Fuchs, 2017). Lastly, empathy extends beyond individuals, encompassing interactions with environments, and is influenced by factors such as immediate social context, interaction history, and communication tools and artifacts (Newen et al., 2018). In short, empathy is not confined to our brain, but depends on an embodied agent (living body and lived body) coupled and embedded within a social environment (Tanaka, 2015). This means that empathy is not simply an isolated cognitive or emotional process, but rather develops within a continuously changing context where both the individuals and their environment interact and mutually influence each other.

Within the 5E framework, the primary experience of sensing another is experienced *directly* as an *embodied cognitive agent* in a *shared context,* where a bodily/affective component is intertwined with cognitive processes (Gallagher, 2012; Fuchs, 2017). This view highlights three main points. Firstly, it directly signifies that the other is experienced without the need to use theoretical inferences or self-simulation, as classical theories claim (Gallagher, 2012; Tanaka, 2015; Fuchs, 2017). Secondly, embodied cognitive agents emphasize that others do not appear to us as mere physical entities or as a hidden mind, but as a whole being where the mind is embodied (Gallagher, 2012; Tanaka, 2015; Fuchs, 2017). Lastly, a shared context refers to the fact that the other embodied agent is not a physical entity in a void, but rather a living body embedded in the world (Tanaka, 2015).

Therefore, in the 5E approach, the other’s bodily expression appears to us as meaningful affect and actions, that express their intentions, needs, and goals in a shared context (Gallagher, 2012; Fuchs, 2017). In this process, our body responds and resonates with the other’s movements, postures, and affective states. This body resonance occurs at a physiological level (living body) and at a subjective level (lived body), where two component of bodily resonance is manifested: an affective dimension (the body is affected by events through bodily sensations) and an e-motive dimension (the body tends to act through body movement) (Fuchs, 2017). Body resonance is not simply a matter of being affected and expressed, it also involves a dynamic process of coordination and synchronization between our bodily responses and those of other people (Lindblom, 2020). For example, research has shown that during natural social interactions, participants unconsciously coordinate their movements and expressions such as facial expressions, vocalizations, and posture (Marsh et al., 2009). This coordination has been linked to empathy, pro-social behavior, and cooperation. Moreover, a meta-analysis found that being in synchrony with others can enhance cooperation, perceived social bonding, partner perception, and affect (Mogan et al., 2017).

Conversely, subtle adjustments in our posture, gesture, and movement generate changes in how both agents are affected, creating a complex cycle of perception-action between the two or more embodied agents (Fuchs, 2013, 2017). In addition, these embodied adjustments affect our emotional engagement and perception of the other being and also encompass meaningful action toward others (Fuchs, 2013, 2017). Fuchs describes that processes as a part of interbodily resonance, which means the embodied agents become parts of a dynamic sensorimotor and interaffective system that connects their bodies by reciprocal movement and mutual coordination (Fuchs, 2017). Thus, from a basic empathic process (e.g., empathy for pain) to a more complex one (e.g., intergroup empathy), the basis of empathy is a direct bodily, sensorial, and affective experience in a coordinated process of two or more embodied agents (Colombetti, 2003; Thompson, 2005; Colombetti and Thompson, 2008). This approach does not reject another cognitive process in empathy but highlights the need for the cognitive ability to imagine how others feel and react, and employ perspective-taking or imaginative transposition in the more complex situation (for details see Fuchs, 2013). Those processes are accompanied by the self-other metaperspective which is the ability to freely move between an embodied egocentric empathy (the other in the self) and heterocentric visuospatial perspective (the self in the other) without losing the own bodily self-consciousness (Fuchs, 2015; Thirioux et al., 2016).

**Figure 3:**
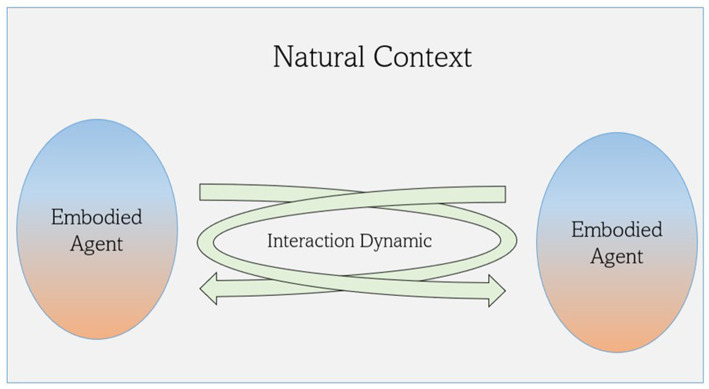
A 5E understanding of Empathy. The examination of empathy highlights the reciprocal influences between embodied agents within their experiential and neurophysiological dimensions embedded in a natural shared context. The arrows represent interaction dynamics encompassing a direct sensorimotor and interaffective coordination between these agents, known as interbodily resonance. This cycle also encompasses the reciprocal exchange of affect and expression, shaping both one’s own experience and the experience of another. This process emphasizes the notion that another being’s “mind” is embodied, and it is through their embodied expression that we can attain a fundamental understanding of their experience.

Overall, the 5E approach offers a comprehensive view of empathy as a direct bodily, sensorial, and affective experience in a shared context. Through the interbodily cycle, embodied agents become part of a dynamic sensorimotor and interaffective system that connects their bodies by reciprocal movement and mutual coordination (Figure 3 adapted from Martínez-Pernía et al., 2023b). In the next section, we will develop a suitable methodology for translating those insights into an ecological research environment.

## 4. Methodological progression based on the 5E approach for a comprehensive study of empathy

When considering the broad construct of empathy coming from 5E approaches, the importance of the mutual influences of embodiment, context, and interaction with others becomes highlighted. This warrants further examination that, currently, exceeds the limitations brought on by traditional laboratory-based studies. However, by transitioning to an interbodily interactive setting, we can unlock a unique opportunity to explore rich intercorporeal dynamics that are embedded in a shared world. This approach can advance our understanding of empathy by delving deeper into the holistic nature of this construct, and by developing new perspectives, models, and theories that are more closely aligned with real-life experiences. The aim is to enhance ecological validity and improve subjective understanding of human behavior in real-world contexts. To achieve this, we propose a methodological approach that capitalizes on multidimensional data (prioritizing high complexity, ecological validity, and behavioral and cognitive degrees of freedom). In this section, we present a novel multimodal neurophysiological approach employing multiple mobile neurophysiological measurements (embodied empathy) combined with rigorous phenomenological examinations of subjective experiences (empathic experience), in an interactive setting and natural context (empathy in an interactive setting).

### 4.1. Embodied empathy

Various novel technologies have been employed to investigate brain activity and body processes in more natural contexts. When combined, they have been called mobile brain/body imaging (MoBI). MoBi is a novel no-invasive mobile technology that allows exploring the body (postural movement, kinematic, muscular activity, eye movement, autonomic correlates) and brain activity in natural settings and during movement (Gramann et al., 2011). MoBi typically involves the use of wireless EEG recording in freely moving individuals and a multimodal approach integrating EEG recordings with measures of muscle activity, body movement, eye movement, and physiological data (heart rate, electrodermal activity, and respiration pattern) (Goldstein et al., 2018; Packheiser et al., 2021). A MoBI approach has been successfully applied to recording EEG and Body in real-life interactions, such as romantic couples interaction (Goldstein et al., 2018; Packheiser et al., 2021), infant-mother interaction (Gramann et al., 2011), classroom dynamics (Dikker et al., 2017), music coordination, and psychotherapy session (Zimmermann et al., 2021). Thus, the MoBI approach has ecological advantages given the possibility of performing measurements in natural interaction, allowing free movement and interaction between embodied agents. In the context of interbodily dynamics, MoBI allows the measurement of the body being affected by another embodied agent and the tendency to act. Following, we will show the main instrument used in MoBi.

#### 4.1.1. Mobile brain recording

In contrast to laboratory EEG, mobile EEG or functional near-infrared spectroscopy (fNIRS), allows the measures of brain responses in real-life situations while the participant stands upright or moves. A novel example of using mobile EEG is the study by Packheiser et al. (2021), which investigated real-life emotions by recording couples at home during interactive activities. Greater Alpha and beta power asymmetry were found during kissing and hugging in the frontal electrodes and less alpha power asymmetry at parieto-occipital electrode sites in the emotional condition compared with the neutral condition (Packheiser et al., 2021). Another study using EEG mobile and eye-tracking devices reveals that in free browsing the display of human face images yielded a face-related N170 ERP (Soto et al., 2018). This face-sensitive ERP component was stronger when viewing disgusted faces than neutral faces (Soto et al., 2018). Using, fNIRS, a study revealed that neural synchrony between two agents in the rTPJ occurred during face-to-face interaction but not during face-blocked interaction, indicating that social gaze may have a significant impact on neural synchrony (Tang et al., 2016). Taken together, these studies open the possibility of studying brain correlates in an embodied interactive context. However, the central tenet in the 5E approach is to couple those correlates with physical/physiological extracranial processes. As we show below, MoBI allows the integration of peripheral measures together with brain signals during unobstructed interactions.

#### 4.1.2. Electromyography

To study body dynamics in natural contexts, it has been used electromyography (EMG) to detect muscle activations. For example, using EMG measurement, Hess and Bourgeois (2010) found that in real interaction between two embodied agents, women smiled more than men, and both genders showed more Duchenne smiles than polite smiles. Furthermore, another study revealed that the muscles of the body respond automatically to the perception of the other’s emotion, without any overt movement. Additionally, the responses of the muscles depend on the type of emotion perceived (Huis in 't Veld et al., 2014).

#### 4.1.3. Posturography

Another instrument to explore embodiment is posturography. Using a measurement of postural responses (center of pressure displacement) with posturography, several studies have shown an approach-withdrawal behavior or freezing responses toward social stimuli (e.g., social threat, emotional faces, affiliative, erotica) (Azevedo et al., 2005; Facchinetti et al., 2006; Roelofs et al., 2010; Gea et al., 2014; Hagenaars et al., 2014; Mouras et al., 2015). Empathy for pain research has shown anteroposterior changes in postural control responses to seeing (passive observations) and imagining (mental simulation) in painful and non-painful scenes (Lelard et al., 2013, 2017; Beaumont et al., 2021). Collectively, these studies highlight the possibility to explore postural movement as a marker of embodiment in a natural posture.

#### 4.1.4. MOCAP

Motion capture is a tool that allows measures of kinematic features of movement (e.g., degree of joints) using cameras and inertial Measurement Units (IMU). In interactive settings, it has been a widely used tool for assessing interbodily kinematics between two agents, allowing for high accuracy compared to observational methods (e.g., behavioral coding). For instance, when assessing infant-adult synchrony dynamics studies found that 14-month-old infants synchronize body dynamics during their face-to-face encounters with an unfamiliar adult (Cuadros et al., 2019). MOCAP allows evaluating subtle changes in kinematic (e.g., joints degrees) behavior in a natural context, thereby, allowing investigation of specific bodily expression in empathy research.

#### 4.1.5. Autonomic physiological responses

Another way to explore the bodily physiology is the study of autonomic physiological responses like heart rate variability, electrodermal activity or respiration (Colombetti, 2013). The exploration of autonomic responses allows the investigation of the first dimension of embodiment where the brain/body interacts to maintain a homeostatic regulation within the human body (Thompson and Varela, 2001; Varela and Thompson, 2003). This organismic regulation has been shown in the induction of heart rate deceleration in children while viewing videos of another’s suffering (Eisenberg et al., 1988, 1991). Furthermore, increased heart rate variability (HRV) was positively related to self-reports of sympathy, compassion, and helping behavior (Eisenberg et al., 1988, 1991). In addition, watching one’s partner perform a physical task that elicits pain and listening to the other’s suffering further increases heart rate and blood pressure compared to a stranger (Monin et al., 2010).

#### 4.1.6. Eye trackers

The use of head-mounted eye trackers has been widely used to assess eye behavior in participants actively engage in natural contexts, such as various sports and naturalistic interactions (Land et al., 1999; Pelz and Canosa, 2001; Vickers, 2007; Hayhoe et al., 2012; Wohltjen and Wheatley, 2021). In social interaction, the study of eye movement has allowed the understanding of the attentional focus, the perception of affective values of another agent (Weng et al., 2018), and the eye movement coordination between embodied agents (Franchak et al., 2011; Barzy et al., 2020). Nevertheless, few empathy studies have explored visual behavior in a natural environment. In natural interaction have been examined visual behavior in mother-infant interaction during free play (Franchak et al., 2011), and in real-life conversations involving subjects with autism (Barzy et al., 2020). On the other hand, lab-based studies have assessed eye movement responses to people suffering (Klin et al., 2002; Joshua and Rossell, 2009; George et al., 2011; Cowan et al., 2014; Weng et al., 2018; Picó et al., 2020). Although few empathy studies evaluate visual behavior in a natural setting, the possibilities offered by these new technologies make it possible to transfer those studies to a real-life situation. These studies support the importance of examining visual behavior in the study of empathy, as a domain that allows us to explore the attention to another’s body signals and the context required to empathize with others.

The evidence presented in this section suggests that the development of robust mobile technology is able to capture body–brain activity as agents actively interact in the natural environment. These tools take advantage of greater embodiment and agentivity and allow exploration of the perception-action cycle of intercorporeal resonance while collecting embodied multimodal information. This illustrates the main difference relative to static measurements; the possibility of whole bodies being affected and expressed in the presence of another agent.

### 4.2. Empathic experience

As previously mentioned, the 5E approach considers conscious lived experience as an irreducible phenomenon (Varela, 1996). The scientific methodology of the 5E approach emphasizes the necessity to explore the phenomenological experience in depth (Varela et al., 1991; Petitmengin, 2006; Thompson, 2007; Colombetti, 2013; Petitmengin et al., 2018; Stilwell and Harman, 2021). In addition, from a more subtle embodied approach of empathy models such as embodied simulation theory (Uithol and Gallese, 2015) and perception-action model (de Waal and Preston, 2017) to a more radical 5E (Gallagher, 2012), highlights the importance to incorporate phenomenological description in the study of intersubjectivity.

As discussed in the previous section, the self-report questionnaires are limited to examining the lived experience in depth. Nevertheless, rigorous methods to explore lived experience through phenomenological interviews have recently been proposed, such as Descriptive Experience Sampling (for details see Krumm and Hurlburt, 2021), micro-phenomenological interview (MPI) (Petitmengin, 2006), the descriptive phenomenological psychological method or Giorgi’s method (Giorgi, 2009), the experimental phenomenological method (Martínez-Pernía, 2022), and PRISMA method (De Jaegher et al., 2017), among others. These proposals are rooted in the descriptive phenomenological tradition, whose goal is to reach an understanding of the structures of human experience (Petitmengin, 2006; Giorgi, 2009; Olivares et al., 2015; Englander and Morley, 2021). Phenomenological methods have been used in cognitive science reports as a single source of information. Thus far, several studies have shown that the description of lived experience can be accurate and also very detailed, allowing for a better understanding of phenomena such as meditative states (Berkovich-Ohana et al., 2013; Nave et al., 2021), awareness in sleep (Alcaraz-Sánchez et al., 2022), temporal variation of emotional state (Depraz et al., 2017), and visual attention (Lachaux et al., 2000), among others (for a review see Berkovich-Ohana et al., 2020).

Conversely, few studies have studied the lived experience in an intersubjective context. For example, using Ollagnier-Beldame and Coupé (2019) explored the lived experience of being with others for the first time in an ecological setting. The finding revealed different experiential modalities and a sense of agency. In another study, lived experience in a classical empathy for pain paradigm was studied using the experimental phenomenological method (Martínez-Pernía et al., 2023b). The results show a multiplicity of bodily sensations, negative emotions, involuntary kinesthetic sensations, and different motivations to act (Martínez-Pernía et al., 2023b). Also, two experiential structures were found: self-centered empathy and other-centered empathy. Both structures distinguish each other by the main direction of attention (toward self versus toward others) and the motivation to act (protective versus prosocial motivation) (Martínez-Pernía et al., 2023b).

Collectively, these studies reveal a promising method to study phenomenology in empathy research. Overall, this method highlights the need for considering phenomenological experience as an essential toolkit to explore deeply the human being based on the 5E approach. Thereby, the inclusion of accurate and detailed phenomenological reports allows for exploring the implicit or unobservable aspect of the embodied agents’ experience, which is critical in a subjective phenomenon such as empathy.

### 4.3. Empathy in an interactive setting

An embodied person is always in an environment where sense-making is shaped by a person’s relationship and interactions with their physical and sociocultural environment (Newen et al., 2018). In empathy, the 5E perspective highlights that the other always appears to us as being with meaningful actions embedded in a context (Gallagher, 2012; Fuchs, 2017). The other person is manifested as an embodied agent “being in the world” (Merleau-Ponty, 1945), and the empathizer’s agent is also another “being in the world.” The world is shared when agents are immersed in real-time and the interactions are reciprocal, such that one agent’s expression affects the other and vice versa (Tanaka, 2015; Fuchs, 2017). These ontological claims, in methodological terms, require a research program where the agents of a study are participants in social and embodied interaction feeling engaged in a shared experience.

In laboratory and natural-environment interactive settings, at least five ways to address live interbodily social interaction have been examined by integrating MoBI and phenomenological interviews. The first refers to the measurement of a single agent while interacting with another, which is the common setting approach in interbodily interactions. This is exemplified in one study that examined real-life emotions by recording brain activation using mobile EEG in a single person at home during couple interactions (kissing, hugging, and emotional speech) (Packheiser et al., 2021).

The second method involves recording brain–body activity from two or more agents in a shared context (e.g., Cheng et al., 2015; Jiang et al., 2015; Baker et al., 2016; Ciaramidaro et al., 2018). This experimental setting allows hyperscanning of the brain–body mechanisms underlying intersubject dynamics (Hari et al., 2013). Studies on empathy have explored physiological and brain-to-brain coupling during the administration of heat pain stimuli in romantic partners, revealing increased physiological and brain synchrony, as well as the analgesic effect of touch modulated by empathy (Goldstein et al., 2016, 2018; Goldstein et al., 2017). Dikker et al. (2021) conducted a notable field study on museum visitors, finding a positive relationship between inter-brain coupling and social closeness, personality traits, focus level, and motivation to connect. Hyperscanning has also been used to evaluate psychological interbodily dynamics during movement. For example, in face-to-face psychotherapy sessions with adolescents, higher movement synchrony and positive correlation with therapy outcomes were observed (Zimmermann et al., 2021). Force-measuring platforms have been used to measure postural sway in live interactions (Reynolds and Osler, 2014), and eye-tracking has been employed to explore infant-mother interaction dynamics.

The third method, group hyperscanning, quantifies social effects on human physiology during real-world interactions with multiple embodied agents. For example, a study of collective rituals has revealed synchronized arousal measured by heart rate dynamics between active participants and related spectators (Konvalinka et al., 2011). Another study in a classroom setting found that inter-student brain synchrony predicts social dynamics and class engagement (Dikker et al., 2017).

The fourth method used to explore the interaction in interbodily settings is the study of phenomenological experience in a live setting. For instance, in the intersubjective context, first encounters have been examined (Ollagnier-Beldame and Coupé, 2019), as the perception of people with autism, and the process of thinking in interaction (De Jaegher et al., 2017).

Together, these studies demonstrate a promising approach to investigating empathy in an interactive setting by leveraging mobile technology. This methodology enables us to study empathy in a manner that closely resembles real-world conditions, facilitating the bridging of the gap between empathy research and its application in natural contexts. By recognizing the interactive and embedded nature of empathy, we gain a deeper understanding of its dynamics and generalizability to real-life situations.

## 5. Empirical 5E approach: integrating mobile brain/body imaging and phenomenological data in interactive environments

In the previous section, an embodied, interactive, and phenomenological experimental setting was proposed as a method to study empathy from the 5E approach. Nevertheless, how to integrate the collection of phenomenological experience with brain/body and intercorporeal dynamics remains unclear. Here, we illustrate the Empirical 5E approach (E5E), that is, an integrative scientific method to bridge neurobiological and phenomenological attributes in an interbody interactive setting. Further, we identify the main three challenges and suggest different approaches to address these challenges.

**Figure 4:**
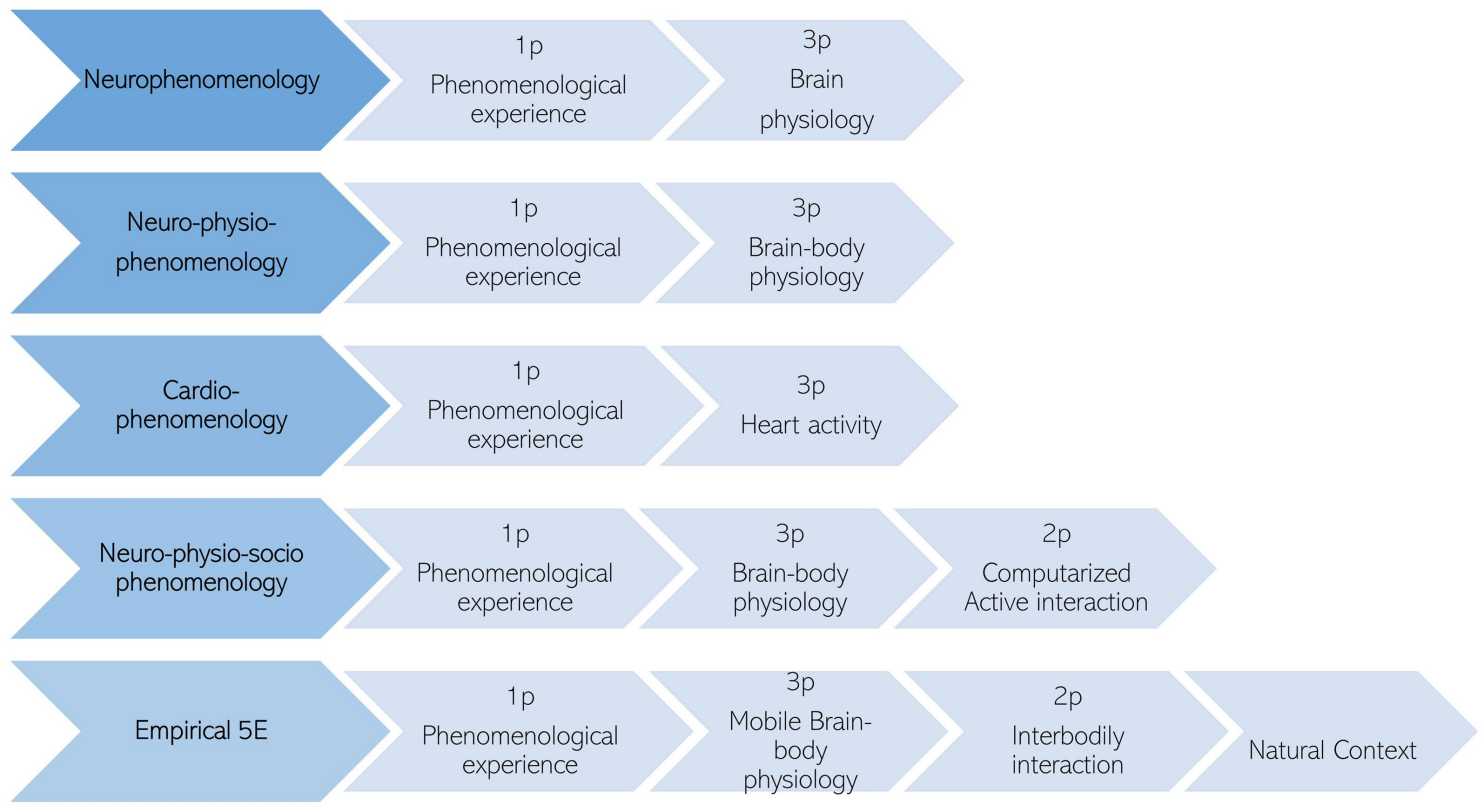
Progression in methodology for bridging gaps: transitioning from subjective experience and neurobiological responses to an approach centered around embodied agents in interactive contexts.

The integration of both biological and phenomenological attributes in a methodological proposal has been the focus of interest of multiple researchers in the past (Figure 4). In 1996 Francisco Varela presented his neurophenomenological research program (NRP), proposing irreducibility in the study of consciousness (Varela, 1996). Through this program, the study of consciousness assumes a double attribute: one of a subjective nature, or “what it’s like” (Nagel, 1974), and another of a physical nature. Neurophenomenology aims to provide a pragmatic methodological framework in which cognitive neuroscience can rigorously integrate a disciplined examination of conscious experience, incorporating the study of phenomenological description and brain activity (Varela, 1996). Various studies have implemented the neurophenomenological approach into research questions, study designs, data analysis, and theory generation with flexible bridges between 1p (first-person or phenomenological experience) and 3p (third-person or brain–body physiology) data (Berkovich-Ohana et al., 2020). Initial ideas of the NRP have been criticized for their brain-centrism, and functionalist view, as well as neglecting the feeling body (Bayne, 2004; Beaton, 2013; Colombetti, 2014). Subsequently, several proposals have been proposed for the refinement of the NRP considering the incorporation of bodily and interactive measurements as an essential tool in the 5E approach (Colombetti, 2014; Depraz and Desmidt, 2019). For instance, cardio-phenomenology highlights the use of heart activity measures synchronizing with phenomenological experience (Depraz and Desmidt, 2019). Another example is neuro-physio-phenomenology which integrates physiological responses, brain responses, and phenomenological data in a research setting (Colombetti, 2014). Another refinement has been proposed by Froese (2015), highlighting the inclusion in a laboratory setting of active engagement with others, namely neuro-physio-socio-phenomenology.

**Figure 5:**
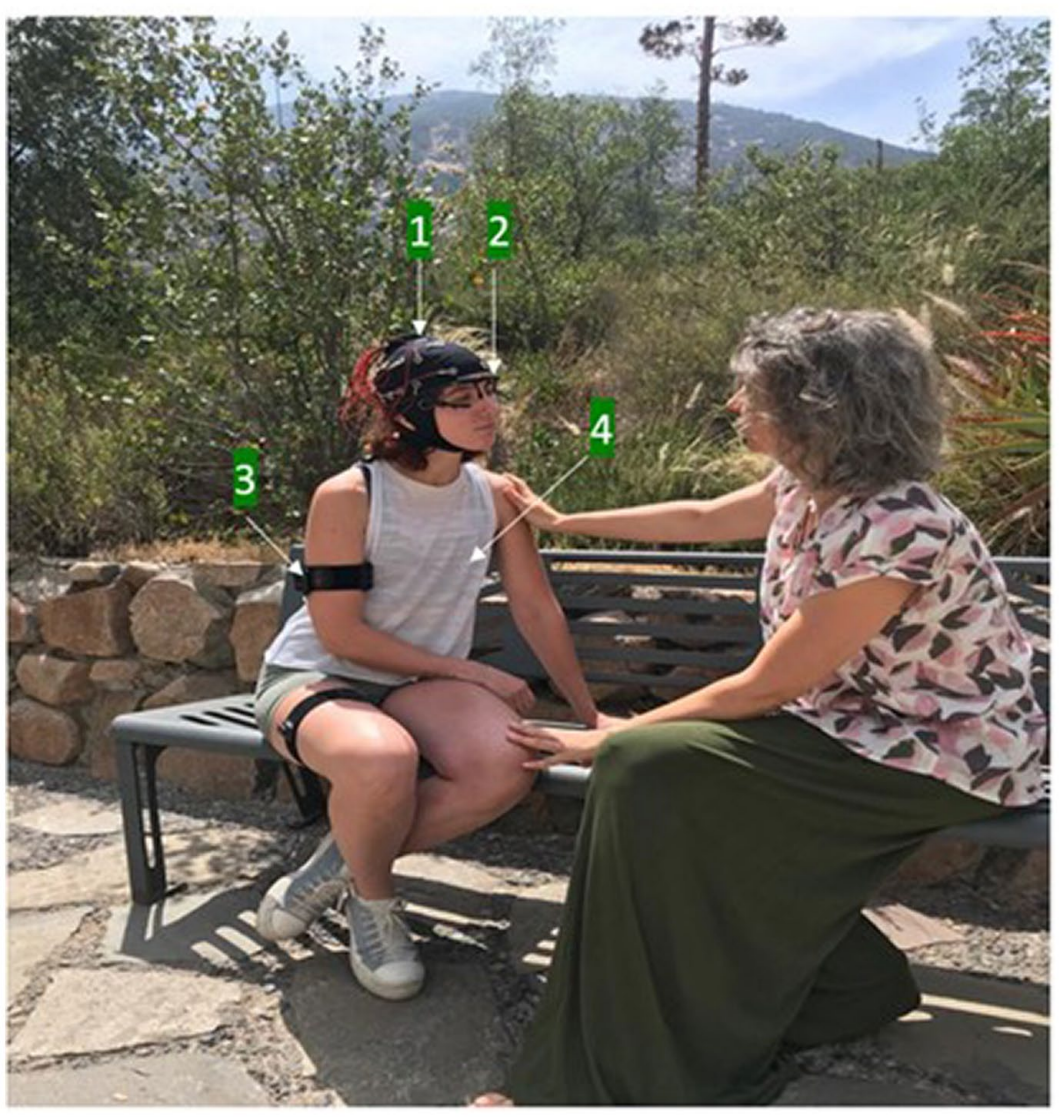
Example of an Empirical 5E approach applied in the empathy study: On the right side, a trained actor shares a personal story about their suffering, employing effective storytelling techniques. On the left side, a participant actively engages in the interaction while being monitored through an unobtrusive MoBi system including mobile EEG (1), eye-tracking (2), IMU (3) and HRV (4) recording. Following the interaction, a phenomenological interview is conducted by a researcher to delve deeper into the participant’s subjective experience.

In alignment with previous theoretical developments, we propose taking one step further in this progression, adding the need to explore the neuro-physio-socio-phenomenology of empathy in more natural settings, exploiting mobile brain and body imaging technology (MoBi), and the phenomenological methodology. In doing so, we present the “Empirical 5E approach” or “E5E framework.” This proposal highlights several technological and methodological advancements for studying empathy in an interactive naturalistic setting using MoBi as well as in-depth phenomenological interviews. By combining these data collection instruments with ecologically valid paradigms, we can bridge the gap between lab-based paradigms and real-world contexts, leading to significant advancements in our understanding of empathy (Figure 5).

Below, we posit four primary ways for examining embodied agents (neurophysiological and phenomenological data) in an interactive context. The aim of these research procedures is to integrate the MoBI technology with phenomenological data in an ecologically valid context of empathic interaction.

1. Clustering embodied agents based on their phenomenological experience in an interactive setting.

The E5E proposal suggests that in order to grasp the intricacy of empathy fully, we need to shift our focus toward exploring the depths of phenomenological experiences. By gaining an understanding of how embodied agents experience interactive dynamics, we may be able to discern various structures of experiences. Consequently, clustering these embodied agents based on their experiential structures could serve as a valuable approach for comparing neurophysiological data among different phenomenological clusters. For instance, Grice-Jackson et al. (2017) developed a methodology that integrated both 1p and 3p analyses of empathy within a controlled laboratory environment. They employed 1P data to establish “phenomenological clusters” based on the conscious bodily experience of vicarious pain (sensory/localized, affective/general), which enabled differentiation based on neural responses. The outcomes present a remarkable illustration of how 1P data can be specifically utilized, as the identified phenomenological clusters demonstrated an explication of neural response variability that would have otherwise been dismissed as mere noise (Lutz et al., 2002). Translating this methodology to a more naturalistic setting offers an opportunity to comprehend the neurophysiological data in individual experiences that are closer to real-world contexts.

2. Understanding the effect of experimental manipulation on embodied agents in an interactive setting.

Significant advancements have been made in empathic understanding through the application of experimental manipulation techniques such as body blocking with Botox (Neal and Chartrand, 2011), inducing first-hand experiences of pain using placebo analgesia (Rütgen et al., 2015a,b; Hartmann et al., 2022), or employing transcranial magnetic stimulation (TMS) (Holbrook et al., 2021). These manipulations have yielded valuable insights into the underlying mechanisms of empathy and its associated neurophysiological processes. However, to further progress the E5E approach, it is essential to explore the impact of specific manipulated factors on empathic experiences and neurophysiological processes in embodied agents within a more naturalistic context.

Taking the manipulation of first-hand experiences of pain using placebo analgesia using in previous studies (Rütgen et al., 2015a,b; Hartmann et al., 2022) as an example, we can outline how the E5E approach would contribute to an expanded understanding of empathy. For instance, the manipulation in a classical lab setting has revealed a decreased level of empathy, as well as a reduction in the affective-motivational neural component of pain (Rütgen et al., 2015b) The authors suggested that analgesics may have an undesired side effect of reducing empathic resonance and concern for others. However, the experience of exploration of these subjective dimensions was limited as also its ecological validity. Therefore, incorporating this specific manipulation in a naturalistic setting using MoBI systems, while also collecting phenomenological experience data, could provide a valuable approach to gaining a better understanding of how first-person experiences influence neurophysiological and phenomenological processes in embodied agents within a more realistic context. This example can be expanded to encompass different validated experimental manipulations commonly employed in laboratory settings.

3. Front-loading phenomenological insights into experimental design.

Multiple authors suggest using phenomenological insights to aid experimental design, aligning with the principles of the E5E approach (Gallagher, 2003; Berkovich-Ohana et al., 2020; Martiny et al., 2021). By incorporating phenomenological analysis, researchers can gain valuable insights that shape the design of experiments in a more naturalistic setting and the formulation of research questions. This approach has been particularly relevant in empathy research, where phenomenological categories such as self-agency, self-location, and visuospatial perspective have informed the development of paradigms for neuroscience studies (Ruby and Decety, 2001; Thirioux et al., 2010, 2014). For instance, two previous studies explored the brain responses in an experimentally manipulated phenomenological dimension of visuospatial perspective (ego-centered and heterocentric) using EEG in an upright position (Thirioux et al., 2010, 2014). By embracing phenomenological insights within the E5E framework, researchers can enhance their understanding of empathy and create more ecologically valid experimental designs.

4. Refinement of temporal analysis based on phenomenological experience.

Phenomenological data can play a vital role in refining or guiding the analysis of physiological data in their temporal dimensions, given the dynamic nature of the interaction and the physiological data and subjective experience involved. Phenomenological interviews can be beneficial in exploring the diachronic dimension of 1P data by asking questions such as: “When did you experience the maximum intensity of distress described earlier?” and “When did the sensation of pressure in your chest begin to diminish?” For instance, Martínez-Pernía et al. (2023b) found that participants experienced the highest level of body resonance when sportspersons fell to the floor (video), providing insight into the temporal dimension and serving as a starting point for physiological analysis. Based in this findings, a study explored the temporal dynamics of physiological data and subjective experience (Troncoso et al., in preparation). By using phenomenological data to refine and guide the analysis of neurobiological data, a more comprehensive understanding of the dynamic nature of empathy can be obtained.

Overall, the E5E approach offers several avenues for advancing our understanding of empathy in interactive contexts. However, like any methodology, it is not without its challenges. These challenges need to be acknowledged and addressed in order to harness the potential of the E5E approach fully. In the next subsection, we describe the main challenges of the E5E approach and suggest different approaches to address them.

### 5.1. Working with the challenges of the E5E approach

The E5E approach proposes a methodological step toward a more naturalistic setting with the current technology available. Unlike traditional laboratory studies, real-world environments introduce a multitude of variables that pose greater difficulty in control, including the multisensorial environment, participant movements, and interpersonal factors, among others. Consequently, adopting an E5E approach necessitates innovative experimental design methods that effectively balance experimental control while accommodating an analytically manageable level of naturalism and uncontrolled influences. This innovative approach encompasses various elements, such as the implementation of multidimensional data collection and analysis, as well as the design of naturalistic paradigms. Numerous authors have extensively discussed the diverse challenges associated with this approach, including finding solutions for data acquisition and synchronization, advancements in mathematical methods for analyzing multidimensional data, and optimizing the signal-to-noise ratio (Ladouce et al., 2017; Parada, 2018; Stangl et al., 2023).

In the following paragraphs, we will delve into three key challenges that arise in relation to experimental design, the collection of phenomenological data, and the limitations of mobile brain imaging when it comes to measuring subcortical data. Likewise, we discuss potential ways to address them using bridges between traditional lab settings and the E5E approach.

5. Improving experimental control in E5E approach

Enhancing experimental control to improve internal validity presents a challenge in naturalistic studies. The goal is to capture real-life contexts with minimal interference or manipulation, also known as the unstructured setting (Parada, 2018). However, a balanced approach that combines elements from traditional structured settings and unstructured settings can be valuable as an initial step. This hybrid setting, called the semi-structured setting, offers some control over variables while preserving the naturalistic study environment (Parada, 2018). In the semi-structured setting, certain aspects of the experimental conditions are standardized or controlled to ensure consistency, while still allowing participants the freedom to engage in behaviors and interactions resembling real-world scenarios. By striking this balance, researchers can enhance experimental control while maintaining the ecological validity necessary for studying naturalistic settings.

One way to achieve this is by employing an empathic manipulation task, similar to those commonly utilized in traditional laboratory studies (e.g., placebo-analgesia, imagining perspective-taking). This task can be based on paradigms previously studied in a laboratory setting and then adapted to suit a naturalistic environment (Martínez-Pernía, 2022). This bridge, also, allows for the extension of knowledge gained from studies conducted in a laboratory setting to more naturalistic contexts, enabling researchers to estimate the generalizability of their findings. An example is manipulating affective perspective-taking by instructing participants to imagine themselves in the situation (self-perspective) or to imagine someone else in the situation (other-perspective) (Decety et al., 2013). Moreover, the manipulation of context through virtual reality (VR) could be replicated in semi-structured natural settings.

Standardizing procedures and protocols across participants and conditions is another critical factor in improving internal validity in natural settings. This could be achieved by establishing a standardized mode of interaction based on previous research (e.g., Packheiser et al., 2021). Although a semi-structured setting can serve as a helpful transition, studies on ecological empathy in natural environments will eventually need to adopt an unstructured approach within real-world settings.

6. Managing the complexity of phenomenological data processing

While classical qualitative methodologies have faced criticisms regarding vulnerability to biases, reliability, and validity; contemporary applications of phenomenological data in cognitive science have employed various strategies to address these concerns. Firstly, the use of guided interviews focusing on pre-reflexive experience helps ensure performative validity (Petitmengin, 2006). Second, researchers employ refined and structured methods of analysis (Petitmengin et al., 2018). Thirdly, innovations in the processes of triangulation and intercoder reliability have also been utilized in phenomenological studies (Nave et al., 2021; Martínez-Pernía et al., 2023b). Lastly, to address the labor-intensive and time-consuming nature of this research, technology (e.g., transcription software) and structured analytical approaches with software support (e.g., Atlas Ti) have been adopted. Interestingly, recent developments of natural language processing artificial intelligence tools could potentially offer support for phenomenological analysis.

7. Studying subcortical areas from the E5E approach

The study of subcortical areas is essential in empathy research as they play a critical role in emotional processing and regulation. Nevertheless, studying subcortical areas using mobile approaches is currently not feasible due to the absence of portable brain imaging technologies with high spatial resolutions. Nonetheless, fMRI studies can still benefit from a more comprehensive empathy view by integrating elements such as context, active interaction and the phenomenological experience. For example, options include using real-time naturalistic stimuli, developing interactive online tasks, and incorporating phenomenological data (Garrison et al., 2013). Another approach to enhance the comprehensive understanding of empathy is to compare data collected in laboratory settings with data collected in the field. This comparison can provide insights into the similarities and differences between the two environments and inform how to adapt laboratory findings to natural settings. For instance, a recent study by Hildebrandt et al. (2021) used fMRI and ecological momentary assessment to investigate how brain activation during an empathy task predicts everyday perspective-taking. This study found that brain activation was predictive of everyday perspective-taking, but not of compassion, distance, or one’s own perspective.

Overall, this section highlights the progression of the E5E approach as a field of study, moving from controlled laboratory settings to examining empathy in more naturalistic environments. Additionally, this view could facilitate the generation of new insights, models, theories, and experimental paradigms. Moreover, employing a naturalistic approach aids in refining our understanding of real-world empathy in health, pathology and promote developing novel interventions in the near future.

### 5.2. Concluding remarks

The field of empathy research in social neuroscience is dedicated to unraveling the intricacies of understanding and experiencing empathy, shedding light on the underlying mechanisms within the brain and body. Traditionally, empathy research has been carried out in well-controlled laboratory settings, which have proven valuable for testing specific hypotheses and gaining insights into fundamental brain functions and their physiological underpinnings. While social neuroscience has laid a foundation for exploring the influences and physiological understanding of complex constructs associated with empathy, it is clear that a more comprehensive and holistic approach is needed to further our understanding of empathy in real-world contexts. Therefore, we propose an embodied, embedded intercorporeal, and phenomenological methodology based on the 5E approach, to advance our comprehension of empathy and fully embrace its inherent complexity. This approach leverages cutting-edge mobile brain/body technology and employs rigorous phenomenological interviews to integrate both the physical and experiential aspects of human beings within interactive settings. By adopting this methodology, we aim to bridge the gap between laboratory-based research and real-world empathic experiences, enabling a deeper understanding of empathy in its natural and dynamic context.

## Data availability statement

The original contributions presented in the study are included in the article/supplementary material, further inquiries can be directed to the corresponding author.

## Ethics statement

Written informed consent was obtained from the individual(s), and minor(s)’ legal guardian/next of kin, for the publication of any potentially identifiable images or data included in this article.

## Author contributions

AT: conceptualization, writing, review, and editing—original draft. DM-P and VS: conceptualization, supervision, writing, review, and editing. AG: constructively reviewed the manuscript. All authors contributed to the article and approved the submitted version.

## Funding

AT and VS was supported by ANID (grant nos. 21220194 and 11221227). DM-P was partially supported by ANID/FONDECYT (11190507). AG was supported by MCIN/AEI/10.13039/501100011033/FEDER, “Una manera de hacer Europa” in the proyect PID2021-127214OB-100.

## Conflict of interest

The authors declare that the research was conducted in the absence of any commercial or financial relationships that could be construed as a potential conflict of interest.

## Publisher’s note

All claims expressed in this article are solely those of the authors and do not necessarily represent those of their affiliated organizations, or those of the publisher, the editors and the reviewers. Any product that may be evaluated in this article, or claim that may be made by its manufacturer, is not guaranteed or endorsed by the publisher.
